# Undulating Seal Whiskers Evolved Optimal Wavelength‐to‐Diameter Ratio for Efficient Reduction in Vortex‐Induced Vibrations

**DOI:** 10.1002/advs.202304304

**Published:** 2023-10-17

**Authors:** Amar M. Kamat, Xingwen Zheng, Julian Bos, Ming Cao, Michael S. Triantafyllou, Ajay Giri Prakash Kottapalli

**Affiliations:** ^1^ Bioinspired MEMS and Biomedical Devices Engineering and Technology Institute Groningen Faculty of Science and Engineering University of Groningen Groningen 9747AG The Netherlands; ^2^ Discrete Technology and Production Automation Group Engineering and Technology Institute Groningen Faculty of Science and Engineering University of Groningen Groningen 9747AG The Netherlands; ^3^ Department of Mechanical Engineering Massachusetts Institute of Technology (MIT) Cambridge MA 02139 USA; ^4^ MIT Sea Grant College Program Massachusetts Institute of Technology (MIT) Cambridge MA 02139 USA

**Keywords:** 3D printing, biological fluid dynamics, biomimetics, microelectromechanical systems (MEMS), seal whiskers, vortex‐induced vibrations

## Abstract

Seals are well‐known for their remarkable hydrodynamic trail‐following capabilities made possible by undulating flow‐sensing whiskers that enable the seals to detect fish swimming as far as 180 m away. In this work, the form‐function relationship in the undulating whiskers of two different phocid seal species, viz. harbor and gray seals, is studied. The geometry and material properties of excised harbor and grey seal whiskers are systematically characterized using blue light 3D scanning, optical and scanning electron microscopy, and nanoindentation. The effect of the undulating geometry on the whiskers’ vibration in uniform water flow is studied using both experimental (piezoelectric MEMS and 3D‐printed piezoresistive sensors developed in‐house) and numerical (finite element method) techniques. The results indicate that the dimensionless ratio of undulation wavelength to mean whisker diameter (*λ*/*D*_m_) in phocid seals may have evolved to be in the optimal range of 4.4–4.6, enabling an order‐of‐magnitude reduction in vortex‐induced vibrations (compared to a similarly‐shaped circular cylinder) and, consequently, an enhanced flow sensing capability with minimal self‐induced noise. The results highlight the importance of the dimensionless *λ*/*D*_m_ ratio in the biomimetic design of seal whisker‐inspired vibration‐resistant structures, such as marine risers and wake detection sensors for submarines.

## Graphical abstract

Phocid seal whiskers possess a unique undulating geometry that reduces vortex‐induced vibrations (VIV) and helps the seal track its prey. In this work, the form‐function relationships in harbor and grey seal whiskers are studied. The results indicate that the wavelength‐to‐mean diameter ratio (*λ*/*D*_m_) of the undulating whiskers may have evolved to possess an optimal value of 4.5 to minimize VIV.

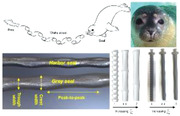

## 1 Introduction

Nature has long served as a model template for the design of many man‐made products and technologies. For instance, marine animals possess a sensory system that has been highly optimized over millions of years of evolution, allowing them to display remarkable survival hydrodynamics.^[^ ^1^ ^]^ Given the fact that most marine animals inhabit conditions comprising murky waters, the presence of sensory organs finely attuned to the tiniest of flow disturbances (often on the order of µm s^−1^) allows them to perform impressive tasks such as hunting prey, escaping from predators, and schooling in synchronization as part of a group in low visibility conditions. The study of the sensory biology of such organisms can inspire elegant solutions for engineering problems. For instance, the lateral line organ of fish, consisting of an array of hair‐like microstructures both on the skin (‘superficial neuromasts’) and under the skin (‘canal neuromasts’), is responsible for helping fish sense flow velocities as low as 18–38 µm s^−1^ in the frequency range of 10–20 Hz.^[^ ^2^, ^3^ ^]^ These neuromast sensors enable the fish to detect objects in their vicinity and effectively map their hydrodynamic surroundings.^[^ ^4^ ^]^ Similarly, crocodiles possess an array of dome‐shaped pressure receptors on their face that are highly sensitive to waves on the water surface (force sensing thresholds ≈0.08 mN and displacements ≈3.9 µm^[^ ^5^ ^]^), allowing them to detect tiny stimuli, e.g. generated by a single water droplet, without the use of vision or audition.^[^ ^6^ ^]^ Finally, seals, the focus of this paper, possess an exquisite capability of long‐distance prey tracking as we will detail.

**Figure 1:**
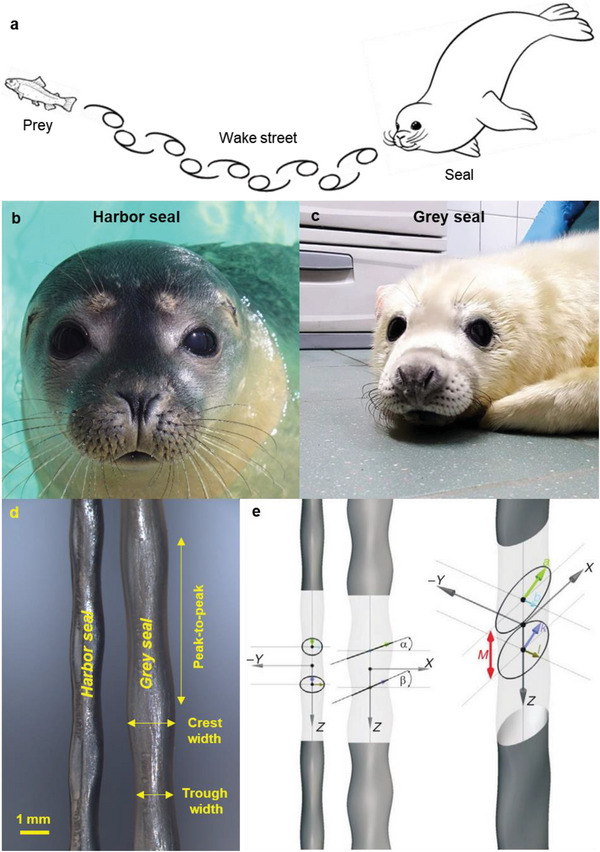
Hydrodynamic trail following using undulating whiskers. a) Schematic of a seal tracking a fish by following its hydrodynamic trail, b) harbor seal, c) grey seal, d) optical micrographs of harbor and grey seal whiskers showing undulations in the whisker geometry along with the measurement parameters used to characterize 2D undulations,^[^ ^10^ ^]^ and e) geometric framework proposed by Hanke et al.^[^ ^14^ ^]^ to describe the 3D undulating structure of the whisker. a) Adapted with permisson. Copyright 2021, ColoringAll.com. B,c) Reproduced with permission. Courtesy of the Zeehondencentrum (Pieterburen, NL). e) Reproduced with permission.^[^ ^14^ ^]^ Copyright 2010, The Company of Biologists.

In their seminal behavioral experiments performed two decades ago, Dehnhardt et al.^[^ ^7^ ^]^ showed that harbor seals (with no visual or auditory cues) were able to follow the hydrodynamic trail of a robotic submarine even when the submarine had a 20 second head start. Using their results, Dehnhardt et al.^[^ ^7^ ^]^ estimated that the seal might be able to track herring swimming up to 180 m away, if the background noise from the surroundings and the aging of the fish trail were assumed to be negligible. Such an impressive ability is made possible by the whiskers on the seal's snout that function not only as active touch receptors but also as ultrasensitive flow sensors, displaying a flow sensing threshold as low as 0.25 mm s^−1^ at a stimulus frequency of 50 Hz.^[^ ^8^ ^]^ Owing to the highly innervated whisker follicles,^[^ ^9^ ^]^ seals are able to detect the flow signatures in the wake of prey that swim ahead of them, even when the prey has swum by several seconds before (**Figure** **1**a). In addition to the high innervation density, several seal species of the *Phocidae* family, such as the harbor seal (*Phoca vitulina*, Figure 1b), grey seal (*Halichoerus grypus*, Figure 1c), harp seal (*Pagophilus groenlandicus*), ringed seal (*Pusa hispida*), and spotted seal (*Phoca largha*),^[^ ^10^ ^]^ display undulations along the length of their whiskers (Figure 1d) that are believed to play a major role in their exceptional hydrodynamic trail tracking capabilities.

Seals swim at speeds on the order of ≈1.3 m s^−1[^ ^11^ ^]^ (Re ≈ 1460 assuming a characteristic dimension of 1 mm for the whisker) and are capable of detecting flow disturbances lower than 1 mm s^−1^.^[^ ^12^ ^]^ This exquisite ability to sense perturbances up to 1000× lower than the seal's swimming speed implies that the whisker must first be able to minimize any vibrations (‘noise’) induced by its own swimming to be able to make itself sensitive to the vortices (‘signal’) left behind in the wake of its prey. A common source of noise for high‐aspect ratio bluff bodies (such as seal whiskers) placed in a uniform flow is the transverse vibration induced by a flow instability in the wake forming behind the body due to flow separation at the sharp curves of the bluff body. The flow instability gives rise to alternating sign vortices on either side of the structure that excite the structure at the same frequency as that of the vortex shedding, causing what is commonly termed as vortex‐induced vibration (VIV). It has been postulated that the undulating geometry of seal whiskers (essentially a tapered cantilever beam having an elliptical cross‐section with periodically varying major and minor axes) is responsible for VIV suppression, allowing the whiskers to experience a significantly high signal‐to‐noise ratio (SNR) in their function as flow sensors.

The premise of biomimetic sensing is predicated upon the study of the underlying physical principles governing the exquisite sensing capabilities of animals, with a view to replicating their morphology, functionality, and performance in man‐made sensors. Unlike the well‐studied fish lateral line system mentioned earlier, the understanding of the seal whisker sensing system is relatively nascent and has thus been the focus of multidisciplinary studies over the past decade,^[^ ^13^ ^]^ with a particular emphasis on the effect of undulations in the whisker morphology on VIV suppression.^[^ ^14^, ^15^, ^16^, ^17^ ^]^ Due to the twin difficulties of limited access to seal whiskers and performing vibration and fluid flow measurements at the small scale of the seal whisker (cross‐sectional dimensions < 1 mm), several researchers also used approaches such as physical modeling (e.g., by performing experiments on scaled‐up whisker‐like structures^[^ ^12^, ^18^, ^19^, ^20^ ^]^) and numerical modeling^[^ ^21^, ^22^, ^23^, ^24^, ^25^ ^]^ to study the effect of undulations on VIV. The insights gleaned from these studies can be valuable in realizing bioinspired engineering applications such as underwater flow sensing,^[^ ^26^ ^]^ biomimetic wind turbine designs with drag force reduction,^[^ ^27^ ^]^ and vibration‐reducing cables in offshore structures.^[^ ^28^ ^]^ In particular, it is of great interest to understand which design parameter (or combination of parameters) of the undulating whisker geometry (Figure 1e) contributes the most to its VIV suppression, since this can have useful consequences for the biomimetic design of low‐noise underwater structures. Further, reliable and accurate knowledge of the geometric and material characteristics of seal whiskers can greatly aid in deciphering the mechanisms via which the whiskers interact with (and become sensitive to) flow vortices during hydrodynamic trail following.^[^ ^13^ ^]^

In this paper, we performed a comprehensive form‐function characterization of grey and harbor seal whiskers with respect to their morphology, material properties, and VIV response in a uniform water flow. The whisker geometry was studied using both 2D and 3D measurement techniques to quantify the undulations and taper in the whiskers. Material properties were measured using first‐of‐its‐kind nanoindentation measurements to estimate the Young's modulus and flexural rigidity of the whiskers. The internal structure of the whiskers was observed using optical and scanning electron microscopy to clearly delineate, for the first time, three different regions in the transverse cross‐section. Further, we compared the vibration characteristics of grey and harbor seal whiskers with those of a similarly‐sized circular cylinder to study the effect of undulations on VIV suppression. Microelectromechanical systems (MEMS) piezoelectric sensors and 3D‐printed piezoresistive sensors were developed to measure the VIV of the whiskers and a comparable circular cylinder in uniform water flow. Similarly, transient fluid‐structure interaction (FSI) simulations were conducted using finite element modeling (FEM) to simulate the vibration response of the whisker and the cylinder in a steady flow, representing an advancement over previous numerical approaches in the literature that only focused on modeling the fluid flow around a rigid whisker structure, neglecting its flexibility. The results of our exhaustive study indicate that the dimensionless ratio of undulation wavelength to mean whisker diameter (*λ*/*D*_m_) in phocid seals may have evolved to be in the optimal range of 4.4–4.6, enabling an order‐of‐magnitude reduction in vortex‐induced vibrations compared to a similarly‐shaped cylinder. This result can help explain the minimal self‐induced noise of seal whiskers that endows phocid seals with an exquisite hydrodynamic trail following capability. Our findings further highlight the importance of the dimensionless *λ*/*D*_m_ ratio in the biomimetic design of seal whisker‐inspired vibration‐resistant structures, such as marine risers, wake detection sensors for submarines, and other underwater structures prone to VIV.

## 2 Results and Discussion

### 2.1 Morphometrics

**Figure 2:**
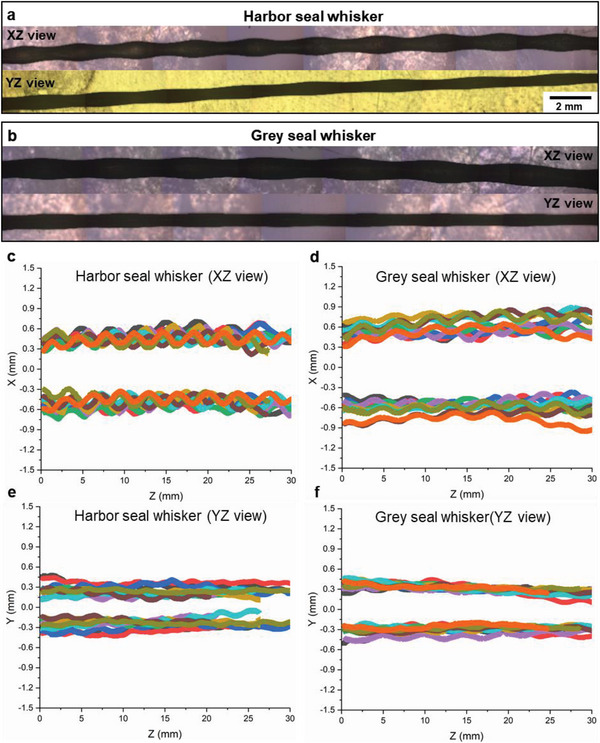
2D morphometric measurements of undulating grey and harbor seal whiskers (ten each). Exemplar stitched optical micrographs of a) harbor seal whiskers and b) grey seal whiskers obtained in both the XZ (showing width) and YZ (showing thickness) views. The scale bar (2 mm) applies to all the micrographs shown in (a) and (b). 2D profiles extracted from the micrographs after image processing: c) ten harbor seal whiskers (XZ view), d) ten grey seal whiskers (XZ view), e) ten harbor seal whiskers (YZ view), and f) ten grey seal whiskers (YZ view).

The geometry of both the harbor and grey seal whiskers features undulations along their length. The whisker is, in essence, a tapered beam with an elliptical cross section whose major and minor axes vary periodically along its length. 2D outlines of ten harbor seal whiskers and ten grey seal whiskers, obtained after optical microscopy and image processing (**Figure** **2**a,b), indicated consistent profiles in the observed length of 30 mm along both the XZ and YZ views as shown in Figure 2c–f. In general, substantial consistency was found between our geometric measurements and previous studies in the literature^[^ ^10^, ^17^, ^29^ ^]^ for the harbor seal whisker. Measurements of the grey seal whisker, on the other hand, were found to be much rarer, with only one study^[^ ^10^ ^]^ that reported morphometrics. Our measurements and their comparison with the literature are summarized in **Table** **1**.

**Table 1:** Comparison of measurements of harbor and grey seal whisker with the literature. The range refers to the standard deviation based on the measurement of 10 whiskers each of harbor and grey seals.

Species	Source	Peak‐to‐peak top [mm]	Peak‐to‐peak bottom [mm]	Crest width [mm]	Trough width [mm]	Crest width / Trough width
Harbor seal	Ref. [10]	3.27 ± 0.39	3.26 ± 0.40	0.92 ± 0.13	0.73 ± 0.12	1.26
	Ref. [17]	3.88 ± 0.45	N/A	1.11 ± 0.88	0.88 ± 0.03	1.27
	Ref. [29]	3.44 ± 0.72	3.45 ± 0.73	1.05 ± 0.24	0.83 ± 0.19	1.26
	This work	3.49 ± 0.33	3.53 ± 0.33	1.17 ± 0.1	0.93 ± 0.19	1.28 ± 0.18
Grey seal	Ref. [10]	3.43 ± 0.38	3.41 ± 0.39	0.76 ± 0.13	0.63 ± 0.11	1.21
	This work	4.36 ± 0.41	4.41 ± 0.42	1.39 ± 0.15	1.23 ± 0.18	1.13 ± 0.14

The measurements shown in Table 1 only provided 2D information of the width of the whisker and neglected its thickness. To capture the 3D undulating morphology of the seal whisker from 2D measurements, Hanke et al.^[^ ^14^ ^]^ proposed a geometric framework comprising a total of seven parameters (*a*, *b, k*, *l*, *M*, *α*, and *β* as defined in Figure 1e). Here, the undulating whisker is approximated as a 3D surface that encloses two periodically recurring ellipses. The primary axis of the initial ellipse (with semi‐major axis denoted as *a* and semi‐minor axis as *b*) is established by connecting adjacent crests along the upper and lower profiles of the whisker. Conversely, the primary axis of the subsequent ellipse (with semi‐major axis referred to as *k* and semi‐minor axis as *l*) is established by connecting adjacent troughs along the upper and lower profiles of the whisker. These two ellipses exhibit inclinations (assumed to be constant in this framework) with respect to the longitudinal axis of the whisker, characterized by angles *α* and *β*, respectively. The alternating repetition of these ellipses (maintaining a separation distance of *M*) along the longitudinal axis can be readily transformed into a 3D surface representation of the whisker using computer‐aided design (CAD) software packages. This geometric framework has been used by researchers for reporting whisker undulation measurements^[^ ^14^, ^29^ ^]^ and to develop 3D models of the seal whisker for experimental^[^ ^12^ ^]^ and numerical^[^ ^23^ ^]^ studies. Using the knowledge of the coordinates of the relevant points from microscopy and image processing, we measured *a*, *k*, *M*, *α*, and *β* from the XZ view, while *b* and *l* were measured from the YZ view according to the definitions given in Figure 1e. **Table** **2** lists the parameters measured in this study and compares them with the measurements of Hanke et al.^[^ ^14^ ^]^ (who used photogrammetry) and Rinehart et al.^[^ ^29^ ^]^ (who used computed tomography scanning) for harbor seal whiskers. The measured parameters for grey seal whiskers are also listed in Table 2; however, for this seal species, no corresponding studies were found in the literature for comparison.

**Table 2:** Comparison of measurements of harbor and grey seal whisker using the geometric framework of Hanke et al.^[^ ^14^ ^]^ The range refers to the standard deviation based on the measurement of 10 whiskers each of harbor and grey seals.

Species	Source	*a* [mm]	*k* [mm]	*b* [mm]	*l* [mm]	*M* [mm]	*α* [°]	*β* [°]	*D_m_* [mm]	$\frac{2M}{D_{m}}$
Harbor seal	Ref. [14]	0.595	0.475	0.24	0.29	0.91^a)^	15.27	17.60	0.8	2.28
	Ref. [29]	0.525 ± 0.118	0.416 ± 0.094	0.178 ± 0.067	0.219 ± 0.083	1.724 ± 0.364	0.299 ± 5.266	1.218 ± 5.838	0.66 ± 0.08	5.26 ± 0.92
	This work	0.58 ± 0.05	0.47 ± 0.09	0.25 ± 0.08	0.3 ± 0.09	1.72 ± 0.2	3.23 ± 13.47	5.07 ± 25.19	0.80 ± 0.14	4.36 ± 0.69
Grey seal	This work	0.7 ± 0.08	0.62 ± 0.09	0.29 ± 0.03	0.33 ± 0.04	2.22 ± 0.27	8.7 ± 16.13	13.24 ± 22.61	0.96 ± 0.07	4.63 ± 0.77

^a)^ It is likely that the value of *M* was mistakenly underreported (by a factor of 2) in Ref.[14] as pointed out by Beem^[^ ^53^ ^]^ in her PhD dissertation.

It must be noted that Hanke et al.’s framework^[^ ^14^ ^]^ assumed constant angles of inclination (*α* and *β*) for the two ellipses shown in Figure 1e. In reality, however, the angles can vary over a wide range.^[^ ^29^ ^]^ As shown in Table 2, our *α* and *β* measurements yielded considerable variations for both seal species (histograms indicating the spread in *α* and *β* values are shown in Figure S1 of the Supporting Information), agreeing with Rinehart et al.’s^[^ ^29^ ^]^ observations. Due to the large spread in the measurements, the concept of an average *α* or *β* is not physically meaningful, but has nonetheless been reported in Table 2 for the sake of completeness. Finally, although the dimensions of the harbor and grey seal whiskers measured in our study (Tables 1 and 2) were different, with the grey seal whisker being wider and thicker than the harbor seal whisker, it is interesting to note that the ratios of wavelength (*λ* ≡ 2*M*) to mean diameter (*D*_m_ ≡ $\frac{a+b+k+l}{2}$) of both species of whiskers were similar, viz. *λ*/*D*_m_ = 4.4 ± 0.7 for the harbor seal whisker and *λ*/ *D*_m_ = 4.6 ± 0.8 for the grey seal whisker. The implications of this ratio on the VIV suppression of whiskers will be discussed later.

**Figure 3:**
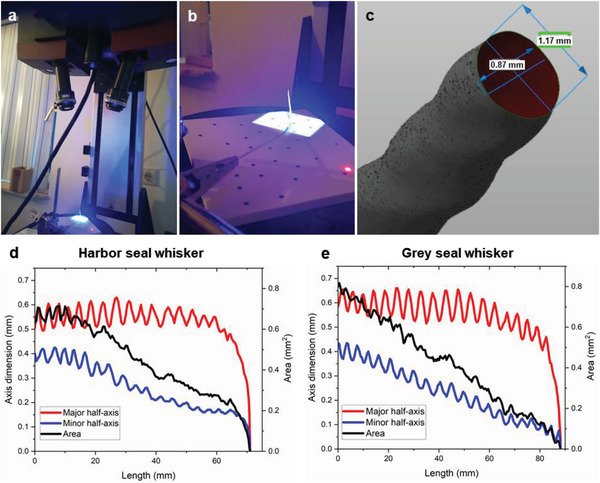
3D morphometric measurements of undulating grey and harbor seal whiskers (one each). a) GOM ATOS III Triple Scan 8M blue light scanning system used for 3D scanning of the whisker, b) close‐up view of the whisker in the process of being scanned, c) the resulting 3D surface model of the whisker and transverse cross‐sectional measurements performed in Autodesk Netfabb. The resulting measurements (semi‐major axis, semi‐minor axis, and cross‐sectional area assuming an elliptical cross‐section) as a function of length were plotted for the d) harbor seal whisker and e) grey seal whisker. Images (a) and (b) courtesy of Lucas van Rennes (GEOSCAN, the Netherlands) and reprinted with permission.

Although the model of Hanke et al.^[^ ^14^ ^]^ allows for an easy construction of a 3D model using only 2D measurements of the whisker along the XZ and YZ views, it is an idealized surface model that neglects several important geometric parameters such as the taper and curvature of whiskers which play a major role in determining their frequency response.^[^ ^30^, ^31^ ^]^ To gain a more complete picture of the whisker morphometry, we also 3D‐scanned one harbor and one grey seal whisker using blue light scanning technology (**Figure** **3**a,b). The resulting digital model (in the form of a .STP file) could be easily viewed in a CAD software such as Autodesk Netfabb which facilitated measurements along successive transverse cross‐sections of the whisker (Figure 3c). A video rendering of the scanned harbor and grey seal whisker models is available in the Supporting Information (Movies S1a and S1b, respectively), showing close‐up 3D views of the undulations along both the width and the thickness of the whiskers. Measurement planes normal to the whisker axis were defined at intervals of every 0.5 mm along the whisker length, and the major and minor axis dimensions (approximating each cross‐section as an ellipse) were measured as a function of the whisker length, allowing us to quantify the taper of the whisker. It must be noted that the major and minor axis dimensions measured in the 3D‐scanned model refer to the transverse cross‐section of the whisker normal to its axis, and are hence different from the parameters (*a*, *b*, *k*, *l*) of Hanke et al.’s^[^ ^14^ ^]^ framework which referred to the dimensions of inclined ellipses (Figure 1e). As seen in Figure 3d,e, the semi‐major axis (i.e., half width) of both the grey and harbor seal whiskers oscillated about a mean value that remained constant (≈0.6 mm for the grey seal and ≈0.55 mm for the harbor seal whisker) for more than half the whisker length, after which it started tapering off rapidly near the whisker tip. On the other hand, the semi‐minor axis (i.e., half thickness) of both whiskers was seen to oscillate about a linearly decreasing mean value, indicating a uniform taper from the whisker base to its tip. The cross‐sectional area also showed a linear decrease from base to tip for both whiskers (Figure 3d,e). The whisker taper in both cases was thus seen to be primarily due to the linear decrease in their thickness, with little contribution from the variation in the width of the whisker. Although the 3D morphometric results presented here were obtained only from one whisker each of a harbor and a grey seal (statistical analysis of five 3D‐scanned harbor and grey seal whiskers is currently underway and will be the subject of future work), the preliminary information reported here can still be used to inform more realistic models of seal whiskers. For instance, Shatz et al.^[^ ^30^ ^]^ calculated the fundamental frequencies of undulating harp seal whiskers by assuming them to be rectangular beams with uniformly tapering width and thickness. The accuracy of such models can be improved by using the morphometric observations from this study, e.g. that the width of the rectangular beam remains constant for over half the whisker length and only starts tapering near the tip of the whisker.

### 2.2 Material Properties

**Figure 4:**
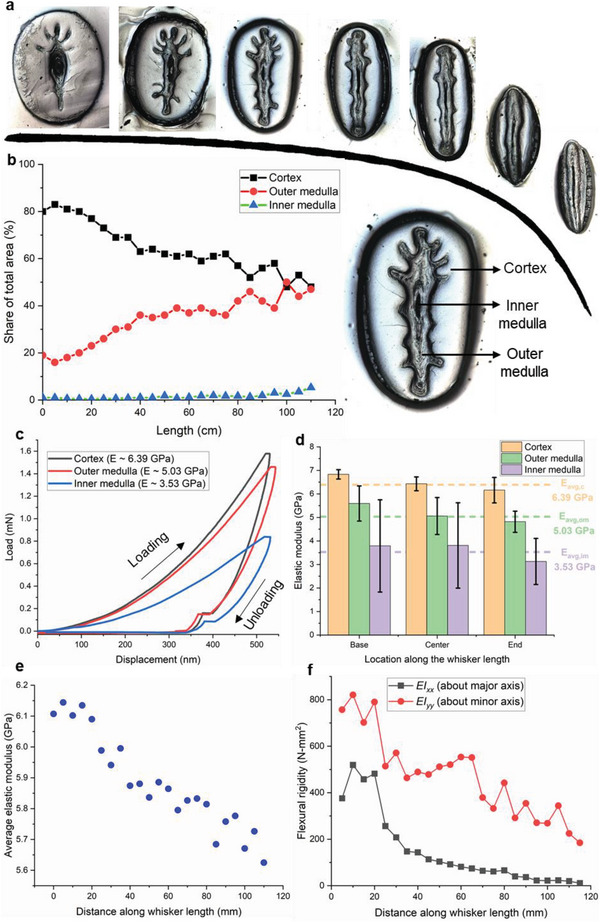
Internal microstructure and properties of the grey seal whisker. a) Optical micrographs of transverse cross sections along the length of the whisker (seven shown out of a total of 24 cross sections), b) identification of three distinct regions, viz. cortex, outer medulla, inner medulla in the transverse cross sections and their corresponding share of the total cross‐sectional area as a function of distance from the proximal end of the whisker (based on a total of 24 transverse cross‐sections). c) Exemplar load‐displacement curves from the nanoindentation tests for the cortex, inner and outer medullar regions, d) Young's modulus values for the cortex, inner and outer medullar regions calculated from the nanoindentation tests at three different locations along the whisker length. e) Average Young's modulus and f) flexural rigidity values calculated along the whisker length.

The polished transverse cross‐section of the grey seal whisker revealed an internal microstructure comprising three distinct regions distinguishable under the optical microscope (**Figure** **4**a,b): the outermost cortex region resembled a smooth shell, the outer medulla appeared darker and comprised an oval region with multiple offshoots, and the inner medulla appeared at the center of the cross‐section and appeared darkest under the optical microscope. A detailed discussion on the internal structure can be found in the Supporting Information Text and Figure S2 (Supporting Infomation). Nanoindentation tests (Figure 4c) revealed a gradation in the Young's modulus values along the radial direction of the elliptical cross‐section. The outermost cortex region was found to be the stiffest (6.39 ± 0.46 GPa), followed by the outer medullar region (5.03 ± 0.67 GPa). The inner medullar region was almost half as stiff as the cortex (3.53 ± 1.57 GPa). Interestingly, the standard deviation in the modulus values of the inner medulla was observed to be much higher, with some tests resulting in elastic moduli as low as 0.4 GPa (near the geometric center of the cross‐section), suggesting the possibility of soft tissue in this region. Further, as seen in Figure 4d, a slight decrease in the moduli was observed for all three regions from the proximal tip to the distal tip. The average Young's modulus average exhibited a slight decrease (from 6.1 GPa at the proximal end to 5.6 GPa at the distal end) over the length of the whisker as shown in Figure 4e. The comparison of our measurements with the aforementioned literature data is summarized in **Table** **3**. Although the spread in the reported Young's modulus values in the literature is high, this is not unexpected given the variety of methods (with their concomitant assumptions) employed in the literature, as listed in Table 3. For instance, the measurement of *E* using a tensile test, implemented either using DMA^[^ ^32^ ^]^ or microtesting,^[^ ^30^ ^]^ is complicated by the fact that a whisker is not shaped like a typical tensile sample, thus making it difficult to convert the measured force into stress due to the non‐uniformity of the cross‐sectional area. Similarly, point load bending^[^ ^31^ ^]^ employed to calculate flexural rigidity (*EI*) and elastic modulus (*E*) is an indirect testing method entailing many assumptions (e.g., approximating the whisker geometry as cylindrical with no undulations or taper in order to use elastic beam bending theory) that can introduce errors in the measured properties. The nanoindentation technique employed in this study is a more direct measurement technique that affords higher resolution both within a cross‐section and along the whisker length. This occurs, however, at the cost of destroying the whisker sample through sectioning and polishing. Finally, we note that there also exists a wide variance in the reported Young's modulus values for the relatively more well‐studied rat whisker, with *E* values ranging from 3–4 GPa^[^ ^33^, ^34^ ^]^ to 7.36 GPa^[^ ^35^ ^]^ in the literature.

**Table 3:** Comparison of measured mechanical properties of seal whiskers with the literature.

Source	Seal species	*E* [GPa]	*EI*_xx_ [N−mm^2^]	*EI*_yy_ [N−mm^2^]	Method of measurement
Ref. [32]	Harbor	2 – 5.5 (distal – proximal)	0 – 1800 (distal – proximal)	N/A	DMA (0.001% and 0.005% strain at different frequencies)
Ref. [31]	Grey	5.9 – 16.5	28 – 34	95 – 98	Point load bending test
Ref. [30]	Harp	1.8 – 3.3	N/A	N/A	Tensile test (microtester)
This work	Grey	5.6 – 6.1 (distal – proximal)	10 – 519 (distal – proximal)	184 – 820 (distal – proximal)	Nanoindentation

An important parameter with respect to the flow sensing performance of the whisker is its flexural rigidity (*EI*), also referred to as bending stiffness. This parameter determines the whisker's bending response due to a tactile and/or hydrodynamic stimulus, and is plotted in Figure 4f as a function of distance along the whisker length (see Materials and Methods for more details). As expected, the rigidity was much lesser around the major axis owing to the elliptical shape of the whisker, allowing easier bending about the major (*XX*) than the minor (*YY*) axis. Interestingly, although Equation (2) (see Experimental Section) suggests that the effective flexural rigidity (plotted in Figure 4f) contains contributions from all the three regions, viz. the cortex, outer medulla, and inner medulla, it was found that the flexural rigidity of the cortex was the major contributor (up to 98%) to the overall rigidity about the whisker major axis, suggesting that the outer and inner medullar regions did not have a significant effect on the overall bending behavior of the whisker about its major axis. The flexural rigidity about both axes decreased along the length of the whisker due to its taper. However, the decrease in *EI_xx_* (indicating resistance to bending about the major axis) was more drastic, reducing by a factor of 5 within only 50% of the whisker length, than the decrease in *EI_yy_* (indicating resistance to bending about the minor axis) which was more gradual in nature. The slightly lower rigidity within the initial 10 mm length of the whisker can be attributed to the fact that this portion is typically embedded within the seal muzzle and thus reinforced by the surrounding tissue and the follicle‐sinus complex.^[^ ^9^ ^]^

Due to similarities in functionality and performance, seal whiskers are often compared to rat whiskers. A major difference that can be surmised from the preceding discussion is in the morphology of the two whiskers — while rat whiskers are smaller and have a conical shape (length ≈10–50 mm, base diameter ≈20–50 µm, and tip diameter ≈5 µm),^[^ ^36^ ^]^ (phocid) seal whiskers tend to be flattened (i.e., elliptical in cross‐section) and contain 3D undulations along their lengths. The morphological differences exist due to the differences in the nature of loading experienced by the whiskers. While rats use their whiskers for object localization and texture recognition (resulting in point loading at the whisker tip), the function of seal whiskers is geared toward both tactile sensing and hydrodynamic trail following. The latter entails a combination of two different kinds of loading on the whisker: a distributed static load caused by the drag force due to the seal's swimming velocity, and a dynamic concentrated load due to the vortices in the wake of, for instance, prey swimming ahead of it. Although no direct in situ observations of the whisker orientation have been made when the seal is in the process of hunting for prey (Hanke et al.^[^ ^14^ ^]^ mounted a camera on a harbor seal and observed whisker position and movement but not orientation), a whisker is often assumed to be oriented such that its angle of attack (AOA), defined as the angle between the major axis of the elliptical whisker and the velocity of the seal's swimming motion, is 0° to minimize the drag force and VIV.^[^ ^14^, ^17^, ^37^ ^]^ In the AOA = 0° orientation, successive vortical stimuli from the wake of the escaping prey then excite the whisker along the direction of its minor axis, causing the whisker to bend about its major (*XX*) axis. Beem and Triantafyllou^[^ ^12^ ^]^ conducted experiments to study the interactions of a scaled‐up harbor seal whisker (AOA = 0°) with a vortex generator placed upstream, and used dye visualization to observe a “slaloming” mechanism that allowed the whisker‐like structure to be locked‐in to the vortical frequency by swaying between the alternating vortices in the wake street. As the structure oscillated transversely to the forward motion, it was synchronized to first approach the closest oncoming vortex on one side and was further pulled toward it due to the low pressure associated with a vortex; then, as the whisker progressed forward, it moved sidewise approaching the next vortex on the other side, again pulled by its low‐pressure gradient, and so on. This constituted the mechanism of vibrational amplitude amplification and hence energy extraction. Such “wake‐induced vibrations (WIV)”^[^ ^12^ ^]^ allow the seal to be attuned to the tiniest of flow disturbances while following the trail of an escaping prey, even when the prey has swum by several seconds before.^[^ ^7^ ^]^ Seen in the light of this discussion, we propose that the low flexural rigidity about the major axis (*EI*_xx_) shown in Figure 4f imparts greater flexibility to the distal half of the whisker, allowing it to effectively slalom its way through a train of incoming vortices. The resulting WIV's are subsequently transduced into a neural signal at the highly innervated whisker base,^[^ ^9^ ^]^ enabling the exquisite ability of the seal to sense flow disturbances up to 1000× lower than its swimming speed. The role of undulations with respect to VIV of grey and harbor seal whiskers is discussed in the next subsection.

Finally, the density of the whiskers was measured as follows. Ten grey seal whiskers were weighed on a microbalance and their collective volume was subsequently noted by measuring the amount of water displaced by them. The average density (mass divided by volume) was calculated to be 1280 ± 80 kg m^−3^ after repeating the mass and volume measurement five times.

### 2.3 VIV Suppression due to Whisker Undulations

**Figure 5:**
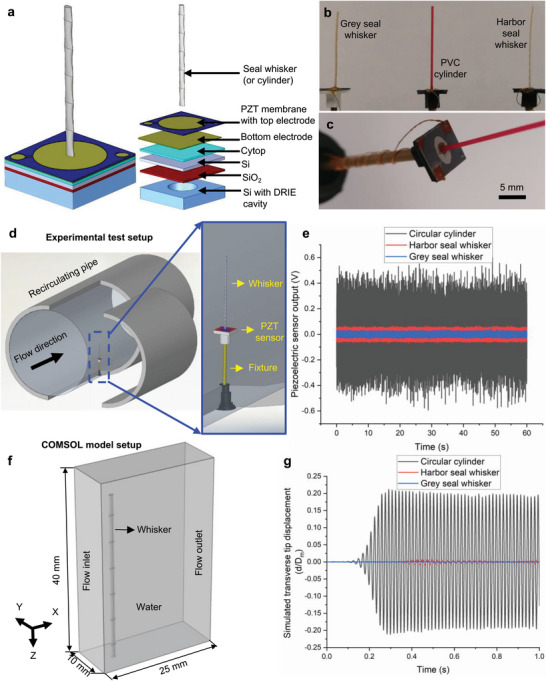
Effect of whisker undulations on VIV. a) Schematic of whisker affixed to the piezoelectric sensor along with the exploded version of the individual components of the MEMS sensor, b) photographs of whiskers and cylinder attached to the piezoelectric MEMS sensor, and c) close‐up photograph showing the MEMS sensor and electrical connections. d) Schematic of experimental setup to test the VIV response of whiskers and a comparable circular cylinder using the MEMS sensor, e) time‐series data recorded by the piezoelectric sensor for the two whiskers and the cylinder for 1 min, f) COMSOL model setup to simulate fluid‐structure interactions of whiskers and cylinder, g) normalized tip displacements (time series data) simulated using the FEM model for the two whiskers and the cylinder.

The undulating whisker morphology of phocid seals, such as the harbor and grey seals, is believed to reduce the VIV phenomenon, resulting in lower noise in the whisker flow sensing system. Prior experiments in the literature conducted with isolated real seal whiskers have produced conflicting results, with some researchers^[^ ^14^, ^15^ ^]^ reporting VIV reduction due to the undulations while others^[^ ^17^ ^]^ finding similar vibration behavior in structures with and without undulations. To study the effect of undulations on the VIV behavior of high‐aspect ratio bluff bodies, we compared the VIV signals of three structures: a harbor seal whisker, a grey seal whisker, and a rigid polyvinylchloride (PVC) cylinder, using a piezoelectric sensor. The material (PVC) and diameter (1 mm) of the cylinder were chosen to maintain similar flexural rigidity and Reynold's number to those of the seal whiskers (more details can be found in Materials and Methods), thus ensuring a fair comparison of VIV responses of the three structures. The seal whisker relies upon the bending moment created by flow disturbances to excite its innervated base and generate a neural signal. This mechanotransduction sensing principle was mimicked by fixing the whisker atop a PZT piezoelectric sensing membrane (**Figure** **5**a–c), wherein the whisker vibrations deformed the membrane and generated a measurable electrical voltage. A piezoelectric MEMS sensor developed in our prior work^[^ ^38^, ^39^, ^40^ ^]^ was used as the sensing base in this study due to its small size (8 mm × 8 mm) and waterproof design. The self‐powered sensor only responded to dynamic forces due to its piezoelectric sensing principle, making it ideal to measure time‐dependent VIV phenomena. The three structures, viz. the harbor seal whisker, grey seal whisker, and the PVC cylinder, were tested at a water flow speed of 0.4 m s^−1^ (Re ≈450 calculated using a characteristic dimension of 1 mm) in a recirculating water tunnel (Figure 5d) and the AOA was maintained at 0° for the two seal whiskers. As seen from the time series data of Figure 5e, the peak‐to‐peak sensor voltage in the case of the PVC cylinder (≈0.8 V) was up to 6× higher than that of the harbor seal whisker (≈0.12 V) and up to 13× higher than that of the grey seal whisker (≈0.06 V), indicating significantly reduced vibrations for the undulating whiskers as compared to the smooth cylinder.

The experiments described above represented an indirect method of deducing VIV through the piezoelectric sensor output. In order to gain more insight into the vibration response of the three structures placed in uniform water flow, an FEM model was constructed in COMSOL Multiphysics (Figure 5f). The simulation results (Figure 5g) reinforced the experimentally observed trend (Figure 5e), namely that the transverse vibration amplitudes for the cylinder were the highest, followed by the harbor and grey seal whiskers, respectively. Interestingly, both the experimental and numerical results suggested that the grey seal whiskers were able to suppress VIV better than the harbor seal whiskers at the tested flow speed. This trend of VIV response (grey seal whisker < harbor seal whisker < cylinder) can be understood in light of a recent study by Lyons et al.^[^ ^23^ ^]^ that used a factorial design of numerical experiments to perform a comprehensive study of the effect of varying geometric parameters describing the harbor seal whisker undulations on *C*_D_ and *C*_L_. Although the researchers^[^ ^23^ ^]^ constructed the “baseline” harbor seal whisker geometry using an erroneous value of wavelength (see footnote to Table 2), the study nonetheless shed light on the relative importance of independent geometric parameters on the resulting VIV performance of the whisker. Lyons et al.^[^ ^23^ ^]^ found that the aspect ratio or slenderness ($\gamma :=\frac{a\cos\alpha +k\cos\beta }{b+l}$), undulation wavelength (λ:=2M), and undulation amplitude along the width ($A_{c}:=\frac{\left|a\cos\alpha -k\cos\beta \right|}{b+l}$) were the (non‐dimensional) parameters that had the largest effect on *C*_L_ and, by extension, on VIV. The sixteen numerical experiments conducted by the researchers provided some indication of how VIV suppression capabilities can change in two undulating structures with different geometric parameters. For instance, there existed a geometry (labeled ‘EL2’^[^ ^23^ ^]^) that had higher *γ*, *λ*, and lower *A*_C_ values compared to the baseline harbor seal whisker geometry and exhibited a lower *C*_L_ than the baseline geometry. Using the definitions and terminology of Lyons et al., our measurements (Tables 1 and 2) revealed that the grey seal whisker also had greater *γ* and *λ* and lower *A*_C_ values compared to the harbor seal whisker (see Supporting Text and Table S1 of the Supporting Information for additional details). Similar to the “EL2” design of Lyons et al., the grey seal whisker also exhibited lower VIV compared to the harbor seal whisker, thus providing qualitative validation of the VIV trend (grey seal whisker < harbor seal whisker < cylinder) observed in our experiments and simulation results (Figure 5e,g).

The presence of undulations in a high‐aspect ratio bluff body, such as a cylinder, disrupts the spatial coherence of the downstream Kármán vortex street^[^ ^12^ ^]^ and thus reduces the VIV experienced by the body. The exact relationship between the geometry of the undulating body and the reduction in VIV is complex and has been a focus of recent studies.^[^ ^23^, ^24^, ^41^ ^]^ For instance, it is of interest to understand which geometric parameters in the whisker's undulating geometry are most relevant to VIV suppression. The manyfold reduction in the VIV response of both harbor and grey seal whiskers compared to a circular cylinder (Figure 5e,g) hinted that the whisker undulations might be optimized for VIV suppression. Interestingly, although the dimensions of the harbor and grey seal whiskers are clearly different (see Tables 1 and 2), both undulation geometries displayed similar *λ*/*D*_m_ values, viz. 4.4 and 4.6, respectively. Further, the *λ*/*D*_m_ values reported in the literature for other phocid seal species, e.g. 4.55 (after correcting for the error mentioned in the footnote of Table 2) for harbor seal whiskers^[^ ^14^ ^]^ and 4.6 for elephant seal whiskers,^[^ ^29^ ^]^ are also in the vicinity of our *λ*/*D*_m_ measurements for grey and harbor seal whiskers, suggesting the importance of the dimensionless *λ*/*D*_m_ ratio toward VIV suppression.

**Figure 6:**
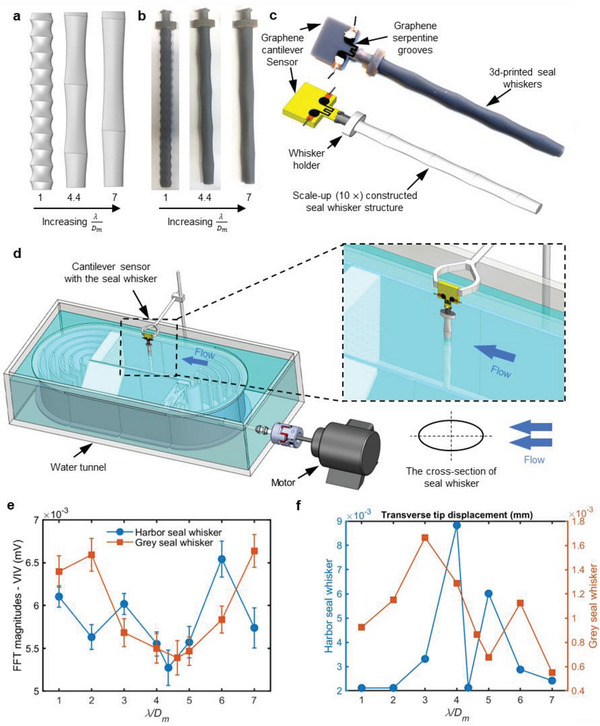
The importance of the *λ*/*D*_m_ ratio in VIV suppression. a) Schematic of whisker‐like undulating structures having three different *λ*/*D*_m_ ratios, b) 3D‐printed whisker structures having three different *λ*/*D*_m_ ratios. c) Assembly of the whisker‐like structure and the 3D‐printed piezoresistive cantilever sensor with GNP strain gauge. experimental setup to test the VIV response of whiskers and a comparable circular cylinder using the MEMS sensor. d) Experimental setup to measure VIV of whiskers having different *λ*/*D*_m_ ratios in a recirculating water flume. VIV response as a function of *λ*/*D*_m_ ratios determined e) experimentally using piezoresistive cantilever sensor and f) via COMSOL FSI model, offering evidence that *λ*/*D*_m_ ≈ 4.4–4.6 represents an optimal ratio for minimum self‐induced noise due to VIV for both harbor and grey seal whiskers.

To explore this hypothesis, we tested the VIV response of 3D‐printed whisker‐like geometries (scaled up 10×) of varying *λ*/*D*_m_ ratios for both harbor and grey seal whiskers. The experiments were conducted in a recirculating water flume (Re ≈1350–1600, similar to the Re experienced by real seal whiskers during foraging), as shown in **Figure** **6**a–d. The whisker structures were mounted on a 3D‐printed piezoresistive cantilever sensor featuring a serpentine graphene nanoplatelet (GNP) strain gauge near its fixed end (Figure 6a,b). The harbor seal whisker geometry (*λ*/*D*_m_ = 4.4) and grey seal whisker geometry (*λ*/*D*_m_ = 4.6) were used as the baseline models respectively, while the other models were constructed by varying the wavelength to generate a total of eight undulating whisker‐like models with the *λ*/*D*_m_ ratio varying from 1 to 7 for each species. Three representative examples are shown in Figure 6a,b pertaining to the harbor seal whisker study. The resulting VIV response (as measured by the graphene‐based cantilever sensor in mV) as a function of *λ*/*D*_m_ (Figure 6e) displayed a local minimum around *λ*/*D*_m_ ≈ 4.4–4.6 for both the harbor and grey seal whiskers. The “optimal *λ*/*D*_m_ ratio” hypothesis was also tested using the FSI model described earlier, wherein similar numerical experiments were conducted to gauge the VIV response (nondimensional tip displacement, *d*/*D*_m_) of whisker structures of varying *λ*/*D*_m_ ratios (Figure 6f). Both the harbor and grey seal whisker curves (Figure 6f) displayed a similar qualitative trend: a local maximum around *λ*/*D*_m_ ≈ 3–4, a local minimum around *λ*/*D*_m_ ≈ 4.4–5, followed by a local maximum again around *λ*/*D*_m_ ≈ 5–6. The qualitative differences in the experimental (Figure 6e) and FEM (Figure 6f) results can be attributed to the differences in how the experiments and FEM models were set up: the experiments (with scaled‐up whiskers) were conducted at Re ≈ 1350–1600 while the simulations were at Re ≈ 135; the 3D‐printed whiskers were partially submerged in the water due to physical constraints, whereas the whisker models were completely submerged in the water domain in the simulations; and so on.

Remarkably, both our experiments and numerical FSI simulations indicated that the *λ*/*D*_m_ ratio of 4.4–4.6, *viz*. the *λ*/*D*_m_ ratio possessed by harbor and grey seal whiskers respectively, was optimal for VIV suppression. Undulating structures are known to disrupt the 2D wake structure (comprising spanwise vortices as experienced by a smooth cylinder) via the production of streamwise vorticity components, resulting in a 3D wake structure. It is probable that at the optimal *λ*/*D*_m_ ratio of 4.4–4.6, the streamwise vorticity components generated at the saddle and nodal planes of the undulating geometry are ideally situated for distorting the spanwise vorticity and maintaining the stability of the 3D wake structure far downstream of the undulating structure. In other words, the interplay between neighboring streamwise vortices at the optimal *λ*/*D*_m_ ratio prevent the spanwise vortex sheets from curling up into mature vortex structures.^[^ ^42^ ^]^ The resulting increase in the vortex formation length can significantly reduce VIV of the whisker structure. A complete mechanistic explanation of this optimality is outside the scope of the current work, and is the subject of current and future work.

Further, our results are in broad agreement with comparable numerical^[^ ^41^, ^42^ ^]^ and experimental^[^ ^43^ ^]^ studies conducted in the literature. As an example, Chen et al.^[^ ^43^ ^]^ recently reported that for harbor seal whisker‐inspired structures (scaled up 70×), an optimal *λ*/*D*_m_ ≈ 4–5 resulted in a 93% reduction in the RMS of the aerodynamic lift coefficient in wind tunnel tests (Re = 38000). Our numerical and experimental data, combined with the fact that similar *λ*/*D*_m_ ratios (in the range 4.4–4.6) have been observed in the whiskers of other phocid seals,^[^ ^14^, ^29^ ^]^ provide strong evidence that the undulating whisker geometry may have been optimized by the process of evolution to minimize VIV‐generated noise, allowing the phocid seal to become finely attuned to the hydrodynamic signals in the wake of escaping prey. These novel findings also highlight the importance of the nondimensional *λ*/*D_m_* ratio in the design of VIV‐resistant bioinspired structures for engineering applications in the future.

It must be noted that the undulating geometry of phocid seal whiskers is comprised of many other geometric factors, in addition to the *λ*/*D*_m_ ratio. It is, for instance, of great interest to understand the functional role of the ellipse inclinations (*α* and *β*) in the whisker geometry, since this staggered waviness is unique to the phocid seal whisker. Further, VIV suppression is one half of the story, and the reaction of the whisker to the wake of the escaping prey (e.g., by a frequency lock‐in with the dominant frequencies of the wake^[^ ^13^ ^]^) to maximize its “wake‐induced vibration” is a key consideration in understanding the evolutionary benefits of the phocid seal whisker structure – this will be the focus of a forthcoming publication.

## 3 Conclusion

The results presented in this paper offer experimental and numerical evidence to underscore the crucial role played by the undulating seal whisker's *λ*/*D*_m_ ratio in the seal's exceptional hydrodynamic trail following feats. Further, the geometric and material property measurements reported here are expected to contribute toward more accurate and comprehensive biomechanical models of the grey and harbor seal whiskers, which will help shed light on the FSI mechanisms that enable the ultrahigh flow sensitivity exhibited by seal whiskers. The identification of *λ*/*D*_m_ as an important dimensionless design parameter for VIV suppression will also help guide the design of bioinspired vibration‐resistant structures and high signal‐to‐noise ratio flow sensors for engineering applications.

## 4 Experimental Section

### Morphometry of Seal Whiskers

Multiple whiskers were excised during the necropsy of deceased grey (two: one male and one female) and harbor (four: three female and one male) seals at the Zeehondencentrum (Pieterburen, the Netherlands). The harbor seals were puppies (age ≈ 10 days to 1–2 years) while the grey seals were adults. Ten whiskers each from both the grey and harbor seals were used for the geometric measurements, and were chosen based on similarity in lengths to ensure meaningful statistical data, since seal whiskers usually span a range of lengths depending upon their position on the seal's muzzle. The whiskers were fixed on to a microscopic glass slide using putty and observed under an optical microscope (Olympus VANOX‐T AH‐2) at a 25× magnification. Micrographs were obtained along two views according to the coordinate system defined in Figure 1e: the XZ view (when the wider dimension of the whisker was parallel to the microscopic slide) and the YZ view (when the narrower dimension of the whisker was parallel to the microscopic slide). Since a seal whisker usually exhibits curvature, the whole length of the whisker typically does not lie in a single plane. Hence, measurements were only conducted on the middle “planar” portion of whiskers encompassing around eight undulating wavelengths (≈30–35 mm length) per whisker. Successive optical micrographs in this portion were obtained along the length of the whiskers and later stitched together using the Adobe Illustrator CC 2018 software. The micrograph was analyzed using an open‐source image processing software (Fiji^[^ ^44^ ^]^) which automatically identified the 2D coordinates of all the local maxima and minima of the undulations.

For the 3D measurements, two representative seal whiskers (one each from grey and harbor seals) were 3D scanned using the GOM ATOS III Triple Scan 8M blue light scanning system. The scanner featured an 8‐megapixel dual camera system, with a 90 mm lens for the cameras and a 120 mm projector lens. The machine was used in the “SO MV60” preset configuration that entailed a scan volume of 60 mm × 45 mm × 35 mm and a measurement accuracy of 17 µm. A thin coating (≈10 µm) of chalk spray was applied to the whisker using an airbrush prior to the 3D scanning to enhance its reflectance. 0.4 mm photogrammetric targets were used. The GOM ATOS Professional software was used to create the main meshing per scan and per side using photogrammetry, and the software Geomagic Wrap was used to assemble these sections into one model. The 3D scanning and reverse engineering processes were performed by a local company (GEOSCAN, the Netherlands), resulting in a digital computer‐aided design (CAD) model of the harbor and grey seal whiskers.

### Internal Structure and Material Properties

A grey seal whisker was sectioned into twenty‐four parts (each ≈5 mm long) and the cross sections were cold‐mounted in a transparent epoxy resin (EpoFix, Streurs). The resin was allowed to cure overnight at room temperature which ensured that the whiskers were not subjected to any high temperature or pressure typically associated with sample mounting processes. The transverse cross sections were then ground with successively finer sandpaper and polished with diamond paste (Struers) to a final roughness of ≈1 µm. Optical (Olympus VANOX‐T AH‐2) and scanning electron (Philips ESEM‐XL30) micrographs were obtained to observe the internal microstructure of the whiskers at magnifications of 50–200×. Further, nine polished cross sections along the whisker length (three near the whisker base, three in the middle, and three near the whisker tip) were subjected to nanoindentation testing (MTS Nanoindenter XP) to measure the Young's modulus. In these tests, the polished cross sections were loaded and unloaded using a Berkovich indenter in the “depth‐controlled” mode up to a depth of 0.5 µm, and the Young's modulus was calculated from the slope of the force‐displacement curve during the unloading stage using the Oliver‐Pharr method.^[^ ^45^ ^]^ A hold time of 3 s was applied at the maximum depth before unloading to allow the biological material to reach equilibrium and to ensure that the biological material's creep rate did not affect the modulus calculation.^[^ ^46^ ^]^ Nanoindentation tests were conducted in three cross‐sections near the proximal end, three near the center, and three near the distal end of the grey seal whisker, resulting in a total of 43 measurements in the cortex, 58 measurements in the outer medulla, and 35 measurements in the inner medulla distributed over nine different polished transverse cross sections of the grey seal whisker.

### Calculation of Average Elastic Modulus and Flexural Rigidity Profiles

Using the area fractions of each region (Figure 4b), the average Young's modulus (*E*_avg_) can be estimated using the rule of mixtures as follows:

(1) $$E_{\mathrm{avg}}=A_{c}\;E_{c}+A_{om}E_{om}+A_{im}E_{im}$$

where *A* is the area fraction, *E* is the Young's modulus, and the subscripts *c*, *om*, and *im* refer to the cortex, outer medullar and inner medullar regions in the whisker cross‐section, respectively. The resulting weighted average indicated a slight decrease (from 6.1 GPa at the proximal end to 5.6 GPa at the distal end) in the Young's modulus over the length of the whisker as shown in Figure 4e.

The effective flexural rigidity, (*EI*)*_eff_*, at a given location along the whisker's length is the cumulative effect of the individual rigidities of the three regions:

(2) $${\left(\mathrm{EI}\right)}_{\mathrm{eff}}={\left(\mathrm{EI}\right)}_{c}+{\left(\mathrm{EI}\right)}_{\mathrm{om}}+{\left(\mathrm{EI}\right)}_{\mathrm{im}}=E_{c}I_{c}+E_{\mathrm{om}}I_{\mathrm{om}}+E_{\mathrm{im}}I_{\mathrm{im}}$$

*E_c_*, *E_om_*, and *E_im_* were obtained from Figure 4d, while the corresponding moments of inertia of the three regions can be calculated by approximating their boundaries as ellipses. If *p* and *q* denote the semi‐major and semi‐minor axes of the elliptical boundaries defining a given region, then the corresponding moments of inertia of the three regions are:

(3a) $$I_{XX,im}=\frac{\pi }{4}\;p_{im}q_{im}^{3}$$

(3b) $$I_{YY,im}=\frac{\pi }{4}\;q_{im}p_{im}^{3}$$

(4a) $$\;I_{XX,om}=\frac{\pi }{4}\;\left(p_{om}q_{om}^{3}-p_{im}q_{im}^{3}\right)$$

(4b) $$I_{YY,om}=\frac{\pi }{4}\;\left(q_{om}p_{om}^{3}-q_{im}p_{im}^{3}\right)$$

(5a) $$I_{XX,c}=\frac{\pi }{4}\;\left(p_{c}q_{c}^{3}-p_{om}q_{om}^{3}\right)$$

(5b) $$\;I_{YY,c}=\frac{\pi }{4}\;\left(q_{c}p_{c}^{3}-q_{om}p_{om}^{3}\right)$$

where the subscripts *XX* and *YY* refer to the moment of inertia computed about the major and minor axes, respectively. Using the major and minor axis measurements of the three regions from the transverse cross‐sections (Figure 4a) and the knowledge of the Young's modulus measurement of each region (Figure 4d), the flexural rigidity about both the major (*XX*) and minor (*YY*) axis of bending was calculated using Equations (2)–(5) and is plotted in Figure 4f as a function of distance along the whisker length.

### Experimental Measurements of VIV Using Piezoelectric MEMS Sensor

The structure of the MEMS sensor comprised a 27 µm thick piezoelectric PZT sensing plate bonded to a 20 µm thick device layer of a silicon on insulator (SOI) wafer with a 0.2 µm oxide layer, resulting in a total thickness of 47.2 µm. The membrane displayed a resonant frequency of 23.3 kHz^[^ ^38^ ^]^ which was orders of magnitude higher than typical seal whisker resonant frequencies that are in the range of 20–200 Hz.^[^ ^30^ ^]^ The sensor was fabricated using MEMS microfabrication processes that included SOI substrate‐PZT layer bonding using spin‐on cytop polymer, chemical‐mechanical‐polishing, sputter deposition of the top and bottom electrodes sandwiching the sensing membrane, patterning of the electrode interconnects and pads, wet etching of the PZT layer to contact the bottom electrode, and releasing the diaphragm structure using the anisotropic deep reactive ion etching (DRIE).^[^ ^38^ ^]^

The 35 mm long whiskers and cylinder were glued on to the piezoelectric membrane using nonconductive epoxy (EPO‐TEK H70E) and subsequently cured in a furnace for 90 min at 80 °C. The whisker vibrations caused buckling of the piezoelectric membrane, generating charges at the top and bottom Au electrodes (Figure 5a). Cladded copper wires were connected to the Au electrodes on the membrane using conductive epoxy (EPOTEK H20E) to establish electrical connections to the external data acquisition circuit (Figure 5c). Finally, nonconductive epoxy was used to seal all the electrical contacts to ensure robust and waterproof connections.

The whisker/cylinder flow sensors were then tested in a recirculating water flow tank that had a circular test section of inner diameter 102 mm (Figure 6a). In order to eliminate the effect of the whisker curvature, 35 mm long straight segments of the whiskers (featuring around 10 undulations) were tested. The material and geometric properties of the PVC cylinder (Young's modulus = 2.43 – 4 GPa^[^ ^47^ ^]^ and diameter = 1 mm) resulted in a flexural rigidity of 120 – 190 N‐mm^2^ that was similar to the rigidity of the whiskers (Figure 4f) tested in the experiment. Moreover, the characteristic dimension of the cylinder (1 mm diameter) was close to the mean diameter given in Table 2 (*D*_m_ = 0.80 ±0.14 mm for the harbor seal and *D*_m_ = 0.96 ±0.07 mm for the grey seal whisker), ensuring comparable Reynolds (Re) numbers for all three flow‐structure interactions. The whisker‐on‐sensor system was glued to a 3D printed fixture (Figure 5b,c) that could be easily screwed into an opening in the recirculating water flow tank. A water flow speed of 0.4 m s^−1^ (Re ≈ 450) was chosen since this was high enough to cause downstream vortex shedding and yet low enough to ensure that the structure (whiskers and/or cylinder) did not separate from the sensing membrane. The sensor output was acquired (via the cladded copper wires mentioned above) using the NI‐DAQ USB‐6003 (National Instruments) and recorded in the NI Signal Express software at a sampling frequency of 10 kHz, and a band pass filter between 0–200 Hz was applied to the resulting data to eliminate the high‐frequency noise and focus on the frequency range relevant to seal whisker vibrations.^[^ ^30^ ^]^ Each experiment was repeated around seven times to ensure repeatability in the results.

### Experimental Measurements of Optimal *λ*/*D*_m_ Ratios Using 3D‐Printed Piezoresistive Sensor

To investigate the effect of the *λ*/*D*_m_ ratio on VIV suppression, CAD models of the harbor and grey seal whisker structures were first constructed using the geometric framework proposed by Hanke et al.^[^ ^14^ ^]^ Parameters *a*, *b*, *k*, *l*, *α*, and *β* (Figure 1e and Table 2) were maintained the same, and the parameter *M* (Figure 1e) was changed to obtain whisker‐like structures with varying *λ*/*D*_m_ ratios equaling 1, 2, 3, 4, 4.4 (harbor seal) or 4.6 (grey seal), 5, 6, and 7 (Figure 6a). The whisker models were then scaled up (10×) in dimensions, with the total length of the structure being 150 mm. The eight structures were 3D‐printed using the stereolithography method (Formlabs Form 3, ‘Grey Pro’ material, Figure 6b). The whisker structure to be tested was mounted on the free end of a 3D‐printed cantilever sensor (Figure 6c) that featured a serpentine graphene nanoplatelets (GNP) strain gauge near its fixed end. The details of the design and fabrication of the GNP‐based cantilever sensor and its high gauge factor and stable piezoresistive behavior as measured in the past work.^[^ ^20^, ^48^, ^49^, ^50^ ^]^ To eliminate errors and uncertainty arising out of sensor‐to‐sensor variability, the same cantilever sensor was used for all the tests, and the whisker‐sensor assembly was designed in a way that the whisker to be tested could be easily mounted and dismounted from the cantilever sensor's free end (Figure 6a–c), thus ensuring a fair comparison between the VIV responses across the varying *λ*/*D*_m_ ratios.

During the tests, the GNP‐based cantilever sensor was located above a recirculating water flume (5L Loligo Systems swim tunnel), with the whisker structure immersed into the water (Figure 6d). The whisker was oriented such that its major axis was parallel to the oncoming flow of 0.15 m s^−1^ speed, i.e., at AOA = 0°. The VIV of the whisker excited the GNP‐based cantilever at the same frequency. The GNP sensor output was obtained using a voltage divider circuit equipped with a low‐pass RC filter, and data were sampled at a rate of 5 kHz using the NI‐DAQ USB‐6289 equipment and the NI Signal Express software. The fast Fourier transform (FFT) operation was conducted upon the resulting time series data, and the VIV response of the whisker structure was quantified by noting the dominant peak in the frequency domain, where the magnitude of the peak indicated the severity of the VIV phenomenon for each whisker structure (three examples are shown in Figure S5, Supporting Information). The structures showed a peak at a frequency of ≈3 Hz, agreeing with theoretical estimate for vortex shedding frequency (≈3.15 Hz) computed using $\frac{St\times U_{\infty }}{D}$ in which *St* is the Strouhal number (0.21 for the expected Reynolds number range), *U_∞_* is the free stream velocity (0.15 m s^−1^, Re ≈1350–1600), and *D* is the characteristic cross‐sectional dimension of the scaled‐up whisker (≈10 mm). The test for each whisker structure was repeated ten times, and the spectral peak of the sensor (characterizing the VIV response) was plotted as a function of the *λ*/*D*_m_ ratio as shown in Figure 6e.

### FEM Modeling of VIV Using COMSOL Multiphysics

To gain more insight into the flow‐whisker interactions leading to whisker vibrations and VIV, FEM simulations were conducted using the coupled fluid‐structure interaction (FSI) module of COMSOL Multiphysics by placing a circular cylinder (diameter 0.6 mm), harbor seal whisker model, and grey seal whisker model in a uniform water flow of 0.2 m s^−1^ (Re ≈ 135). The CAD models of the seal whiskers were constructed directly in COMSOL Multiphysics using the framework of Hanke et al.^[^ ^14^ ^]^ and the measurements given in Table 2. Each time‐dependent simulation lasted one second which was long enough to neglect any effect of initial transients. The structures were located in a flow domain (water) of dimensions 40 mm × 25 mm × 10 mm (Figure 6c), and the fixed end of the cantilevered structures was maintained at a distance of 5 mm away from the side walls and the upstream flow inlet. Note that in reality, the proximal end of the whiskers was embedded in soft material, i.e., the seal's muzzle, rather than being rigidly fixed as assumed in the simulations. In the case of the whiskers, the flow direction was along their major axis (i.e., AOA = 0°). A laminar flow model was used to solve the fluid mechanics equations, while linear elasticity was assumed for the deformation of the structures in the coupled FSI model. The wall condition at the structure‐fluid interface was no‐slip, and the surface mesh of the solid structure was made fine to ensure proper representation of surface morphology of the seal whisker.

The flow domain was divided into six subdomains (Figure S3a,b, Supporting Information) that included two rectangular and four trapezoidal regions (inlet and outlet faces are labeled). The trapezoidal subdomains surrounded a rectangular subdomain (3 mm x 3 mm x 30 mm) that contained the structure (i.e., the whiskers or the circular cylinder). This arrangement was chosen to ensure a dense mesh in the fluid domain around the cylinder where the flow separation regions have to be finely resolved. A tetrahedral mesh with a minimum element size of 0.0632 mm and a maximum element size of 0.585 mm was used for the solid domain (i.e., whiskers or cylinder). For the rectangular subdomain surrounding the solid structure, a predefined mesh with a minimum element size of 0.316 mm and a maximum element size of 1.06 mm was used (Figure S3c, Supporting Information). Further, a swept mapped mesh was used for the four trapezoidal domains and the downstream rectangular domain. The total number of elements of the meshed simulation domain was on the order of 10^6^.

The coupled FSI model solved both the governing equations of fluid mechanics (i.e., water flow behavior) and solid mechanics (deformation of structure) together, allowing to directly observe the whisker and cylinder (0.6 mm diameter) displacements in contrast with earlier numerical models in the literature^[^ ^21^, ^22^, ^23^, ^24^ ^]^ that focused only on the fluid behavior while assuming the solid structure to be rigid. The grey and harbor seal CAD models used in the FSI simulation were constructed using the framework of Hanke et al.^[^ ^14^ ^]^ and the morphometric measurements (Table 2). Identical material properties (Young's modulus = 6 GPa, density = 1300 kg/m^3^) were used for all the three solid structures based on the measurements of the grey seal whisker. The three structures were cantilevered at the base and placed in a uniform water flow of 0.2 m s^−1^. The VIV response was observed by plotting the transverse tip displacement normalized by the mean diameter (*d*/*D_m_*) as a function of time for a total simulation time of 1 s (Figure 5g). The simulation showed that all three structures initially bent in the direction of the flow due to the drag force (Figure S4, Supporting Information), after which they started vibrating transversely due to forces exerted by periodic vortex shedding (Movies S2a–c, Supporting Information). Unlike the two whiskers, the cylinder also vibrated along the flow direction at a frequency (122 Hz, Figure S4, Supporting Information) that was almost double the frequency of transverse vibration (62 Hz, Figure 6d) and an amplitude (*d*_f_/*D*_m_ ≈ 0.05, Figure S4, Supporting Information) that was a third of the amplitude of the transverse vibration (*d*/*D_m_* ≈ 0.14, Figure 6d). Such relations between the in‐line and transverse vibrations were remarkably similar to the numbers reported for flexible isolated cylinders placed in uniform flow,^[^ ^51^ ^]^ thus serving as verification of the computational model. Further, the VIV frequency calculated from the simulated tip displacement (≈62 Hz) showed good agreement with the theoretically predicted^[^ ^52^ ^]^ vortex shedding frequency behind the cylinder (≈66 Hz), where the latter was computed using $\frac{St\times U_{\infty }}{D}$ in which *St* is the Strouhal number (0.21 for the expected Reynolds number range), *U_∞_* is the free stream velocity (0.2 m s^−1^), and *D* is the cylinder diameter (0.6 mm).

## Conflict of Interest

The authors declare no conflict of interest.

## Author Contributions

A.M.K. and X.Z. contributed equally to this work. A.M.K. conceptualized and co‐supervised the project, performed the nanoindentation testing and internal structure characterization, conducted data analysis, and wrote the manuscript. X.Z. performed the COMSOL Multiphysics simulations, fabricated the 3D‐printed cantilever sensors, performed the experimental and numerical *λ*/*D*_m_ study, conducted data analysis, and edited the manuscript. J.B. performed the 2D measurements of the whisker geometry, packaged the piezoelectric MEMS sensor and whisker assembly, and conducted VIV suppression testing in the water tunnel. M.C. co‐supervised the project, edited the manuscript, and acquired funding. M.S.T. co‐supervised the project and edited the manuscript. A.G.P.K. conceptualized and co‐supervised the project, fabricated the piezoelectric MEMS sensors, performed internal structure characterization, acquired funding, and edited the manuscript. All the authors contributed to the discussion of results and a critical reading of the manuscript.

## Supporting information

Supporting Information

Supplemental Movie 1a

Supplemental Movie 1b

Supplemental Movie 2a

Supplemental Movie 2b

Supplemental Movie 2c

## Data Availability

The data that support the findings of this study are available in the supplementary material of this article.
