# Supplementary material for: Undulating Seal Whiskers Evolved Optimal Wavelength‐to‐Diameter Ratio for Efficient Reduction in Vortex‐Induced Vibrations
Source: Adv Sci (Weinh). 2023 Oct 17;11(2):2304304. doi: 10.1002/advs.202304304 (PMC10787063; doi:10.1002/advs.202304304)
Supplement: Supplementary file 1 — Supporting Information [file ADVS-11-2304304-s002.pdf]

ADVANCED SCIENCE
Open Access

Supporting Information

for *Adv. Sci.*, DOI 10.1002/advs.202304304

Undulating Seal Whiskers Evolved Optimal Wavelength-to-Diameter Ratio for Efficient Reduction in Vortex-Induced Vibrations

Amar M. Kamat, Xingwen Zheng, Julian Bos, Ming Cao, Michael S. Triantafyllou and Ajay Giri Prakash Kottapalli**

Supplementary Information for

Undulating seal whiskers evolved optimal wavelength-to-diameter ratio for efficient fish wake tracking

Amar M. Kamat[1†], Xingwen Zheng[1†], Julian Bos[1], Ming Cao[2], Michael S. Triantafyllo[3,4], Ajay Giri Prakash Kottapalli[1,4*]*

[1]Bioinspired MEMS and Biomedical Devices, Engineering and Technology Institute Groningen, Faculty of Science and Engineering, University of Groningen, Groningen 9747AG, the Netherlands.

[2]Discrete Technology and Production Automation Group, Engineering and Technology Institute Groningen, Faculty of Science and Engineering, University of Groningen, Groningen 9747AG, the Netherlands.

[3]Department of Mechanical Engineering, Massachusetts Institute of Technology (MIT), Cambridge, Massachusetts 02139, United States.

[4]MIT Sea Grant College Program, Massachusetts Institute of Technology (MIT), Cambridge, Massachusetts 02139, United States.

[*]Corresponding authors: a.m.kamat@rug.nl (AMK) and a.g.p.kottapalli@rug.nl (AGPK)

[†]These authors contributed equally.

This PDF file includes:

Supplementary Text

Figs. S1 to S5

Table S1

Captions for Movies S1a–b and S2a–c

Other Supplementary Materials for this manuscript include the following:

Movies S1a–b and S2a–c

Supplementary Text

Internal structure

The cuticle, which is usually a thin protective coating (~ 10 μm thick) around the whisker, was not clearly visible in the optical micrographs. The area percentages of the three regions (estimated using the freehand selection tool of the Fiji software) differed along the whisker length (Fig. 4b). While the cortex dominated the microstructure (by area %) near the proximal end of the whisker, the cortex and the outer medulla occupied equal areas at the distal tip of the whisker with the inner medullar area being negligible throughout the whisker length. Unlike the rat whisker which is known to have a hollow medullar region extending up to 80 % of its length, optical microscopy did not provide conclusive evidence of a cavity in the inner medullar region of the seal whisker.

To explore further, scanning electron microscopy (SEM) of sectioned cross-sections was conducted (Fig. S2, Supplementary Materials) to observe the inner medullar region more closely. The SE micrographs showed that although the inner medullar region was not hollow, it contained elliptical cavities (arrowed in Fig. S2) approximately 50 μm × 10 μm in dimensions, suggesting the possibility of centrally connected microchannels along the whisker length. Such microchannels along the inner medullar region of the whisker can serve as a route for hormone transfer from the proximal to the distal end of the whisker, since recent reports[1,2] have confirmed the presence of hormones such as cortisol, progesterone and testosterone along the length of seal whiskers. From a biomechanics standpoint, the bending stiffness and the frequency response of the whisker are not likely to be affected by the presence such microchannels due to the comparatively small area occupied by the inner medullar region (Fig. 4b in the main text). The

function of the microchannels can thus be postulated to be biological rather than mechanical in nature.

Comparison of grey and harbor seal whisker geometry and VIV performance

Lyons *et al.*[3] recently conducted a parametric study where they studied the relative effect of several non-dimensional geometric parameters (pertaining to the whisker undulations) to their VIV suppression performance. The non-dimensional parameters (γ, λ, A_T, A_C, ε, and φ) could be easily calculated from the parameters of Hanke *et al.*'s[4] framework (a, b, k, l, M, α, and β) using simple algebraic formulae. γ, λ, and A_C were deemed to have the most effect on VIV suppression. Table S1 shows these three non-dimensional parameters calculated from our measurements (given in Table 2 of the main text) and compares them to two particular whisker designs (out of sixteen) from Lyons *et al*. It can be seen that for certain relations between geometric parameters (e.g., greater γ and λ and lower A_C for EL2 as compared to the baseline model), the VIV performance can be better. Although the absolute values are different, similar trends with respect to the geometric parameters and VIV performance were observed in our work.

Supplementary References

1. Keogh, M. J. *et al.* Reproductive and stress-related hormones in whiskers from two North Pacific phocids: Harbor and ringed seals. *Mar Mamm Sci* **36**, 1322–1333 (2020).
2. Keogh, M. J. *et al.* Whiskers as a novel tissue for tracking reproductive and stress-related hormones in North Pacific otariid pinnipeds. *Conservation Physiology* **9**, (2021).
3. Lyons, K., Murphy, C. T. & Franck, J. A. Flow over seal whiskers: Importance of geometric features for force and frequency response. *PLOS ONE* **15**, e0241142 (2020).
4. Hanke, W. *et al.* Harbor seal vibrissa morphology suppresses vortex-induced vibrations. *Journal of Experimental Biology* **213**, 2665–2672 (2010).

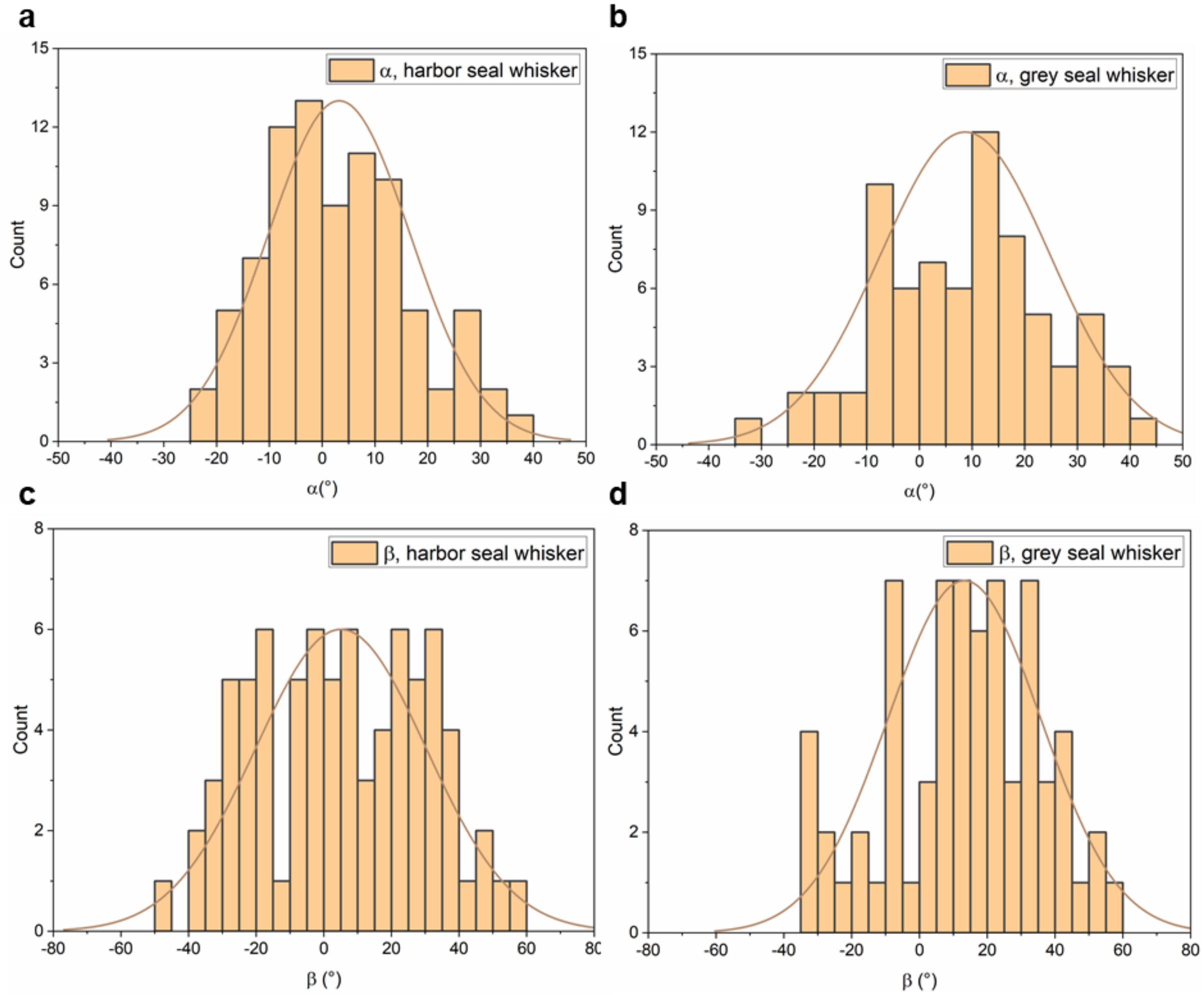

Fig. S1. Histograms, along with the respective normal distribution fits, illustrating the large variance in α and β (defined in Fig. 1d of the main text): a) α for the harbor seal whisker, b) α for the grey seal whisker, c) β for the harbor seal whisker, and d) β for the grey seal whisker.

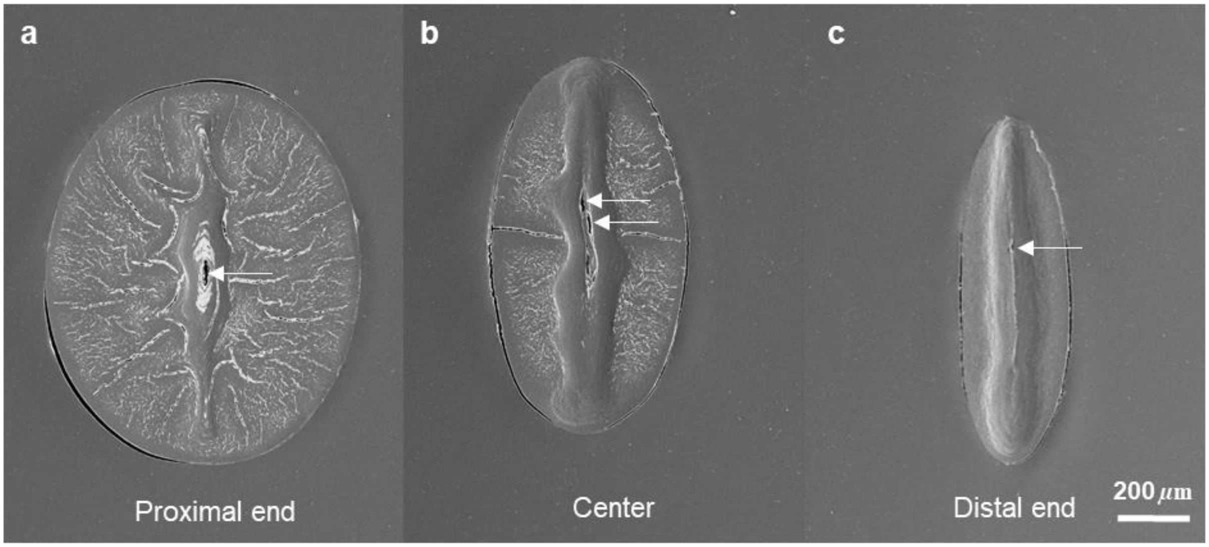

Fig. S2. Scanning electron micrographs of polished grey seal whisker cross-sections at different locations along the whisker: a) near the proximal end, b) at the center of the whisker, and c) near the distal end. Possible microchannels have been indicated by an arrow.

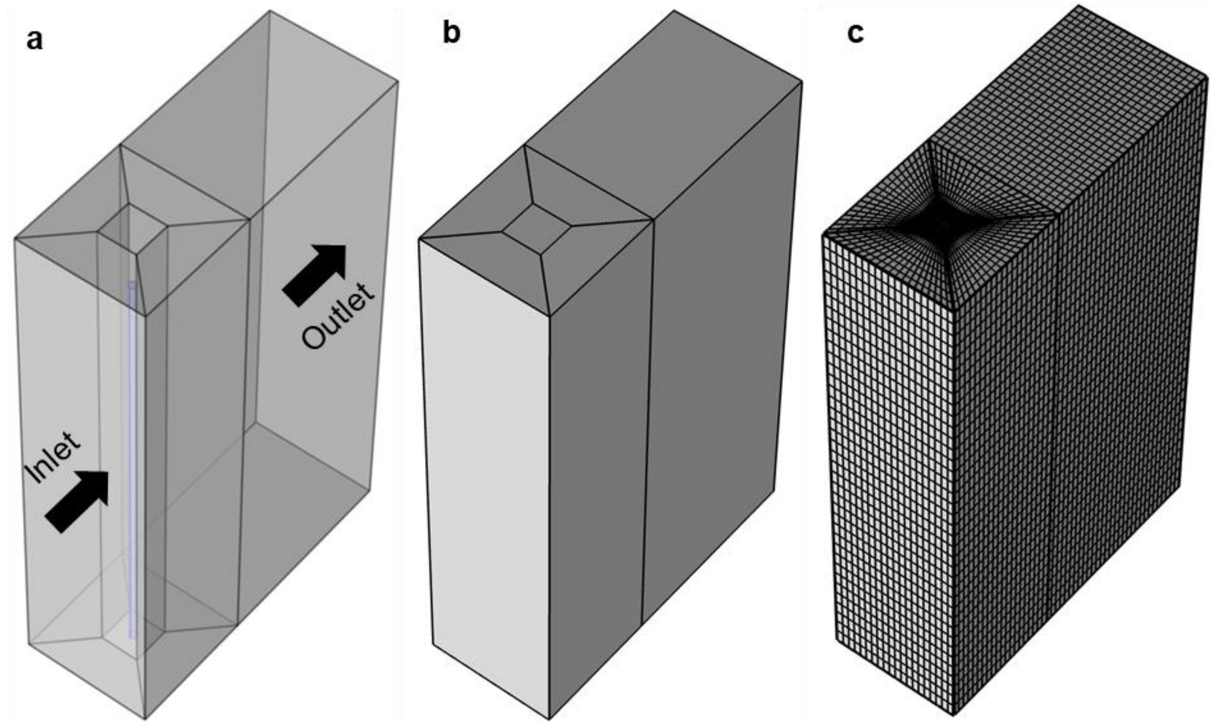

Fig. S3. COMSOL Multiphysics® simulation box for fluid-structure interaction simulations.

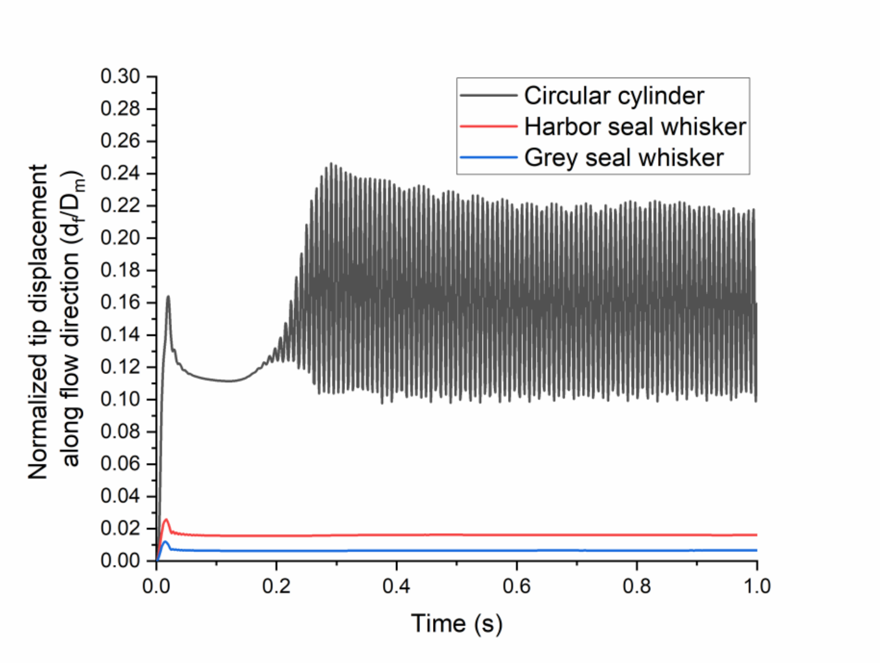

Fig. S4. COMSOL Multiphysics® simulation showing the tip displacement (normalized by diameter) of circular cylinder, grey seal whisker, and harbor seal whisker along the flow direction plotted as a function of time.

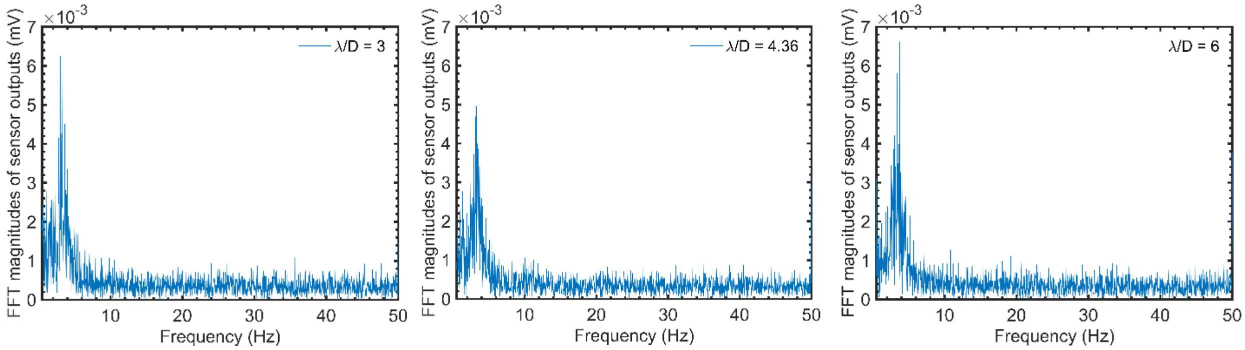

Fig. S5. Exemplar FFT plots obtained from time series data of whisker-like structures, showing the frequency and magnitude of the VIV response for three different λ/D_m ratios.

Table S1. Relations between geometric parameters and VIV performance.

	**Model**	γ	λ	A_C	**VIV performance**
**Lyons *et al.*[21]**	Baseline (based on harbor seal whisker)	3.4	1.9	0.23	Simulated C_L of EL2 lower than that of baseline model
	'EL2'	5	3	0.05	
**This work**	Harbor seal whisker	1.90	6.25	0.20	Grey seal whisker's vibrations lower than harbor seal whisker's (experimental and numerical results)
	Grey seal whisker	2.08	7.16	0.14	

Movies S1a–S2b.
3D rendering in Autodesk® Netfabb® software of scanned a) harbor and b) grey seal whiskers.

Movies S2a–c.
COMSOL Multiphysics® simulation showing VIV of: a) circular cylinder, b) harbor seal whisker, and c) grey seal whisker models placed in a uniform water flow of 0.2 m/s. The streamlines representing water flow run from left to right. The deformations have been scaled up by a factor of 50× in all the videos. The velocity legend shown in (a) is common for all three simulation movies.
